# Mental Health Problems and Their Association With Internet Use in Medical Residents

**DOI:** 10.3389/fpubh.2020.587390

**Published:** 2020-10-21

**Authors:** Tsukasa Ueno, Kazushi Ito, Toshiya Murai, Hironobu Fujiwara

**Affiliations:** ^1^Integrated Clinical Education Center, Kyoto University Hospital, Kyoto, Japan; ^2^Department of Neuropsychiatry, Graduate School of Medicine, Kyoto University, Kyoto, Japan; ^3^Artificial Intelligence Ethics and Society Team, RIKEN Center for Advanced Intelligence Project, Tokyo, Japan

**Keywords:** medical residents, mental health problems, depression, anxiety, self-esteem, internet use

## Abstract

**Objectives:** Mental health problems (MHP) among medical residents are often found in clinical settings and sometimes lead to professional lapses. Evidence suggests that excessive Internet use is associated with MHP. We investigated the MHP of residents (depression, anxiety, and self-esteem) and their association with Internet use using a longitudinal design.

**Methods:** Participants were 208 residents. The General Health Questionnaire (GHQ), Patient Health Questionnaire (PHQ), and the Rosenberg Self-Esteem Scale (RSES) were used to assess anxiety, depression, and self-esteem. The Generalized Problematic Internet Use Scale 2 (GPIUS2) was used to measure Internet use. Data were obtained twice, at baseline and 3 months later when the risk of MHP in residency is highest.

**Results:** Residents with MHP (*N* = 36) had higher GHQ scores than those without MHP (*N* = 172) at follow-up. Residents with MHP had more depression and less self-esteem than those without MHP at baseline and follow-up. GPIUS2 total scores, and scores on the subscale preference for online social communication, were higher in residents with MHP. Preference for online social communication at follow-up was positively correlated with depression at baseline and follow-up, and negatively correlated with self-esteem at follow-up.

**Conclusions:** Depression and self-esteem may predict Internet use when the risk of MHP is greatest for residents, indicating potential risks of excessive Internet use or Internet use as a compensatory coping behavior. Together with depression and self-esteem assessment, Internet use may be a useful index of resident mental health.

## Introduction

Promoting the mental health of medical residents is important for successful learning in daily clinical practice. Evidence suggests that residents in the early phases of their residency are particularly prone to mental health problems (MHP), typically depression and anxiety (1–3) MHP may lead to mental illness and lapses of professionalism (4, 5) that hamper residents' daily clinical performance.

Internet use is a common aspect of daily life. In addition to using the Internet for personal reasons, residents must spend substantial time in the online environment to promote their learning in clinical and academic settings. Although Internet use is useful and safe, excessive Internet use is classed as a behavioral addiction termed “Internet addiction” or “online addiction” (6–8). Excessive Internet use is associated with psychiatric disorders such as depression and anxiety (7, 9) and other related psychological indices, such as low self-esteem (10–13). Thus, excessive Internet use is a behavioral problem that may be associated with subtle signs of MHP in residents. Furthermore, although mental health professionals generally make adequate judgments about MHP, medical educators who are not mental health experts may not. Hence, together with conventional psychological measures, Internet use could function as an index to predict residents' MHP to provide subsequent mental health support. The study aim was to investigate levels of Internet use in residents and the relationship between MHP and Internet use. We hypothesized that MHP, as indicated by depression, anxiety, and low self-esteem, would be associated with a greater degree of Internet use in residents.

## Methods

### Participants

A total of 256 medical residents employed at Kyoto University Hospital from 2015 to 2017 participated in this study. There are two 2-year postgraduate residency program courses in Japan; one is a crossed course in which residents train for 1 year at each of two hospitals, and the other is a continuous course in which residents train at one hospital for 2 years. In this study, first-year residents from a continuous course and second-year residents from a crossed course were recruited. It was assumed that the stress experienced by second-year residents would be very similar to that experienced by first-year residents, in terms of substantial changes in work environment (e.g., changes in patients, workplaces, and medical staff members). Participants had no history of diagnosis of psychiatric illness and no severe physical illnesses that could potentially affect their mental states. Participant demographic characteristics were as follows: subjects with MHP, defined as residents who presented with MHP resulting in their maladaptation at any time during the 2-year residency: age = 26.60 years, standard deviation (SD) = 3.05 years, male/female = 28/16, and residents without MHP: age = 26.40 years, SD = 3.02 years, male/female = 142/72).

The study was approved by the ethics committee of the Kyoto University Graduate School and Faculty of Medicine and was conducted in accordance with the guidelines of the Declaration of Helsinki.

### Presentation of MHP

Subjects with MHP were residents who exhibited anxiety and/or depression and lapses in professionalism, such as unexplained delays or absences, emotional conflicts about other medical staff, difficulties in communication, lack of motivation in learning, and greater number of mistakes in clinical settings. Of the 44 residents with MHP, nine requested individual meetings with us directly in relation to their problems, and 35 were observed by medical staff members to have had problems in clinical settings. A medical educator (psychiatrist, author HF) assessed residents' mental status as part of their mental health management.

### Questionnaires

Questionnaires were distributed at the beginning of the residency (baseline: T1) and at the follow-up 3 months after baseline (T2). The data were primarily used to inform medical educators who interviewed residents or communicated with them to provide mental health care during their residency, and were used in the current study as a retrospective dataset.

### Assessment of General Health and Depression

We used the 12-item General Health Questionnaire (GHQ) (14) to detect signs of depression and anxiety as part of general health, and the nine-item Patient Health Questionnaire (PHQ) (15) to assess the degree of depression. The GHQ-12 has been translated into more than 20 languages and used internationally as a standard screening tool for mental health (14). The PHQ-9 is an appropriate tool to assess symptoms of depression. Each GHQ item is rated on a four-point scale of 0 to 3; higher scores indicate poorer health. The two most commonly used scoring types are the bimodal and the four-point Likert scoring methods; we used the former method (the total score range is 0–12) (16). PHQ responses are on a four-point scale ranging from 0 to 3 (the total score range is 0–27), and standard cutoff score for screening for possible major depression is >10 (15, 17, 18).

### Other Psychological Variables Related to MHP and Excessive Internet Use

The Rosenberg Self-Esteem Scale (RSES) (19, 20) was used to assess the degree of self-esteem. The RSES contains 10 items rated on a four-point scale (the total score range is 10–50). Higher scores indicate higher self-esteem.

### Assessment of Internet Use

#### The Generalized Problematic Internet Use Scale 2 (GPIUS2)

The Generalized Problematic Internet Use Scale 2 (GPIUS2) (21) was used to measure Internet use. The GPIUS2 consists of 15 items rated on an eight-point scale (the total score range is 15–120) to assess the degree of Internet use. The GPIUS2 consists of five subscales: “preference for online social interaction,” “mood regulation,” “compulsive Internet use,” “cognitive preoccupation,” and “negative outcomes.” Each subscale has three items. Scores on an additional “deficient self-regulation” subscale can be calculated by summing CU and CP scores (21). In this study, the combined “deficient self-regulation (SELF)” subscale was used for statistical analysis. Higher scores indicate a higher level of Internet use. We used the Japanese translation of the original scale.

### Statistical Analysis

Demographic data were analyzed using two-tailed *t*-tests or χ^2^ tests. Between-group differences in the data at T1 and T2 were assessed using repeated measures analysis of covariance, with measures repeated at T1 and T2, group as a between-subjects factor, and age and gender as nuisance covariates. *Post-hoc t*-tests were used to compare T1 and T2 scores for each item. Pearson's correlation coefficient or Spearman's rank correlation coefficient was used for the correlational analysis, depending on whether initial exploration suggested normal distributions. Correction for multiple comparisons was not applied to the correlational analyses because of the exploratory nature of the study.

## Results

**Figure 1:**
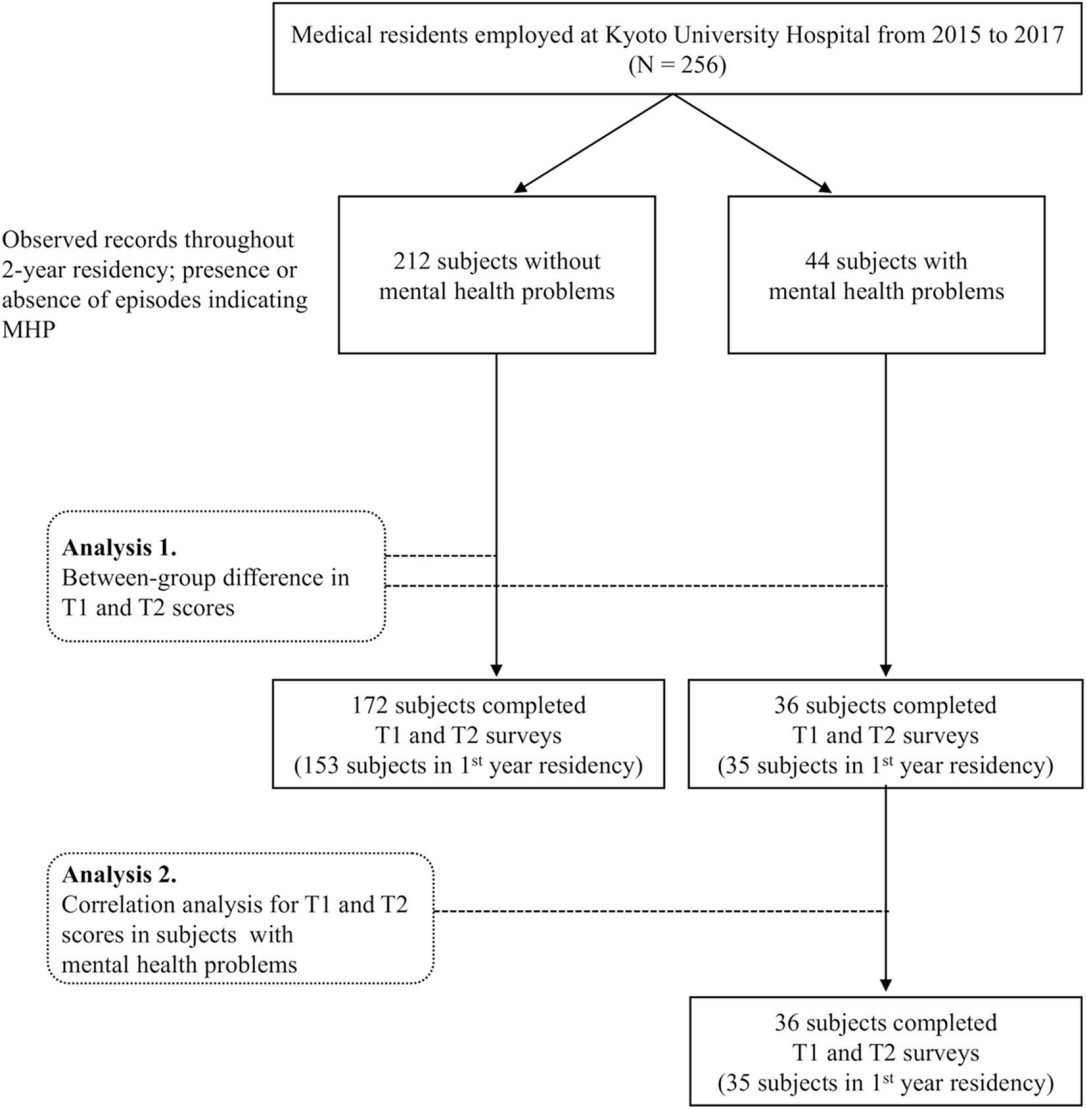
Flow chart of recruitment and analysis.

A retrospective investigation of the observation records of the residents revealed that, of the 256 participating residents, 44 had issues that suggested MHP. Except for one resident who was judged to be clinically depressed, residents with MHP had subclinical levels of anxiety and/or depression resulting in poor adaptation to their residency. A flow chart of the recruitment and analytical process is shown in Figure 1. Of the 256 residents, 208 (36 in residents with MHP and 172 in those without MHP) completed both the T1 and the T2 questionnaires. Therefore, the data for these 208 subjects were used for longitudinal data analyses. In this dataset, the two groups were matched on age and gender: residents with MHP/residents without MHP: age = 26.58/26.40 years, SD = 3.08/3.07 years, *t* = 0.929, *p* = 0.354; male and female = 21 and 15/109, and 63, $\chi_{\left(1\right)}^{2}$ = 0.322, *p* = 0.570). Table 1 shows the descriptive statistics and comparisons of psychological data. GHQ and PHQ scores were within subclinical levels. RSES and GPIUS2 scores were also relatively low, similar to the data of previous studies (21–23).

**Table 1:** Group comparisons between residents with MHP and residents without MHP.

**Independent variables**		**MHP (*N* = 36)**	**No MHP (*N* = 172)**	**Main effect of group**		**Group-by-time interaction**		***Post-hoc*** **test**	
		**M (SD)**	**M (SD)**	***F*_**(1, 206)**_**	***p***	**F**	***p***	***t***	***p***
General Health Questionnaire-12, (GHQ)	T1	4.5 (4.4)	3.3 (4.1)	11.019	0.001^*^	1.161	0.282	1.554	0.122
	T2	4.2 (3.4)	2.0 (2.7)					4.233	<0.001^**^
Patient Health Questionnaire-9 (PHQ)	T1	3.2 (4.6)	1.8 (2.7)	16.749	<0.001^**^	5.338	0.022^*^	2.431	0.016^*^
	T2	3.4 (5.2)	1.0 (1.9)					4.826	<0.001^**^
Rosenberg Self-Esteem Scale (RSES)	T1	24.9 (4.5)	26.6 (4.3)	12.943	<0.001^**^	6.323	0.013^*^	−2.219	0.028^*^
	T2	23.5 (4.6)	27.1 (4.7)					−4.205	<0.001^**^
GPIUS2 Total score	T1	41.6 (19.5)	36.0 (16.0)	4.017	0.046^*^	0.023	0.880	1.838	0.067
	T2	42.0 (20.9)	37.0 (16.8)					1.569	0.118
GPIUS2 Preference for online social interaction	T1T2	7.9 (4.5) 8.2 (4.8)	6.4 (3.6) 6.9 (4.0)	5.159	0.024^*^	0.75	0.785	2.078 1.658	0.039^*^ 0.099
GPIUS2 Mood regulation	T1	9.4 (4.7)	9.4 (4.7)	1.766	0.185	3.764	0.054	0.043	0.966
	T2	9.8 (5.4)	8.1 (4.5)					2.008	0.046^*^
GPIUS2 Deficient self-regulation	T1	16.6 (8.0)	14.6 (7.0)	1.563	0.213	0.497	0.481	1.512	0.132
	T2	17.1 (10.1)	16.3 (7.8)					0.515	0.607
GPIUS2 Negative outcomes	T1	7.0 (4.7)	5.6 (3.6)	0.803	0.371	3.42	0.066	1.999	0.047^*^
	T2	7.4 (5.6)	7.7 (4.5)					−0.377	0.707

MHP, mental health problems; GPIUS2, Generalized Problematic Internet Use Scale 2; SD, standard deviation; T1, Time 1 (baseline); T2, Time 2 (3 months after baseline),

* p < 0.05,

** *p < 0.01*.

### Group Comparisons Between Residents With MHP and Those Without MHP

All group comparison results for each measure are described below, regardless of the statistical significance of the group and group-by-time interaction effects and those of the *post-hoc* analyses, because all group differences were of interest at both T1 and T2. Table 1 shows the results of the group comparisons for each measure.

### General Health (GHQ) Scores

There was a significant main effect of group [*F*_(1, 206)_ = 11.019, *p* = 0.001], but the group-by-time interaction was not significant. A *post-hoc* analysis of variance (ANOVA) showed that GHQ scores were significantly higher in residents with MHP than in those without MHP at T2 (*t* = 4.233, *p* < 0.001).

### Depression (PHQ) Scores

There was a main effect for group [*F*_(1, 206)_ = 16.749, *p* < 0.001], and the group-by-time interaction was also significant (*F* = 5.338, *p* = 0.022). *Post-hoc* analyses showed that the PHQ scores in residents with MHP were higher than those in residents without MHP at T1 (*t* = 2.431, *p* = 0.016) and T2 (*t* = 4.826, *p* < 0.001).

### Self-Esteem (RSES) Scores

There was a main effect for group [*F*_(1, 206)_ = 12.943, *p* < 0.001], and the group-by-time interaction was also significant (*F* = 6.323, *p* = 0.013). The RSES scores were lower in residents with MHP than in those without MHP at T1 (*t* = −2.219, *p* = 0.028) and T2 (*t* = −4.205, *p* < 0.001).

### Problematic Internet Use (GPIUS2) Scores

For GPIUS2 total scores, there was a main effect for group [*F*_(1, 206)_ = 4.017, *p* = 0.046], but no group-by-time interaction. There was also a significant group effect for preference for online social interaction subscale scores [*F*_(1, 206)_ = 5.159, *p* = 0.024], but no group-by-time interaction. *Post-hoc* analyses showed that residents with MHP scored higher than those without MHP on preference for online social interaction at T1 (*t* = 2.078, *p* = 0.039), on negative outcomes at T1 (*t* = 1.999, *p* = 0.047), and on mood regulation at T2 (*t* = 2.008, *p* = 0.046). However, none of the other total or subscale scores differed between the two groups.

### Correlational Analysis

**Figure 2:**
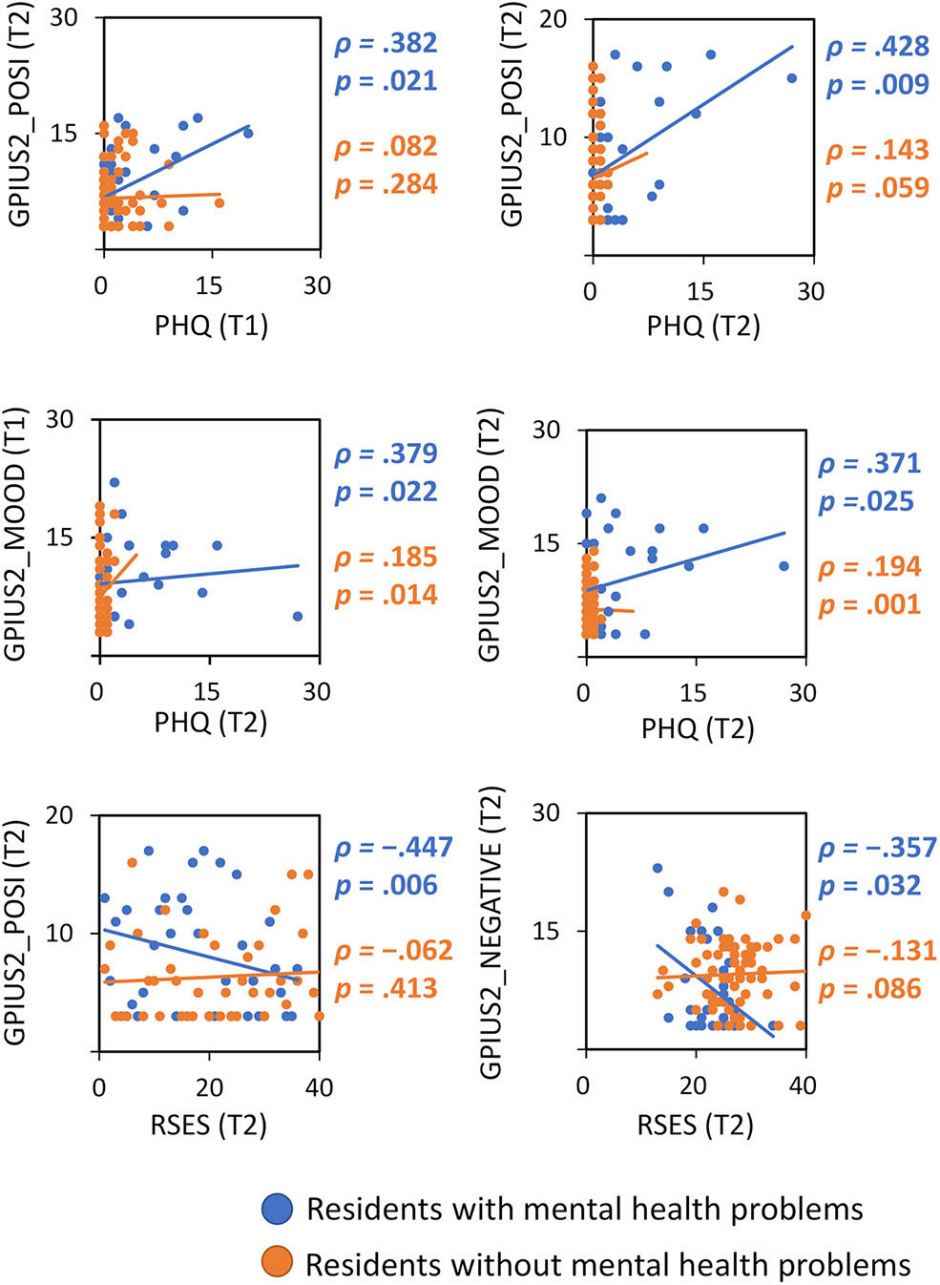
Scatterplots of correlations between Internet use (GPIUS2 score) and other psychological variables in residents with/without mental health problems. PHQ, Patient Health Questionnaire-9; RSES, Rosenberg Self-Esteem Scale; POSI, preference for online social communication; MOOD, mood regulation; NEGATIVE, negative outcomes; T1/T2, time 1 (baseline)/time 2 (3 months after baseline).

Kolmogorov–Smirnov tests showed that none of the correlational analysis variables were normally distributed, so Spearman's rank correlation coefficient was used. For residents with MHP, GPIUS2 mood regulation scores at T1 were positively correlated with PHQ scores at T2. None of the other GPIUS2 total scores or subscale scores at T1 were correlated with GHQ, PHQ, or RSES scores at T1 and T2. For T2, GPIUS2 preference for online social interaction scores were positively correlated with PHQ scores at both T1 (ρ = 0.382, *p* = 0.021) and T2 (ρ = 0.482, *p* = 0.009) and negatively correlated with RSES scores at T2 (ρ = −0.447, *p* = 0.006). GPIUS2 mood regulation scores at T2 were positively correlated with PHQ scores at T2 (ρ = 0.371, *p* = 0.025). GPIUS2 negative outcome scores were negatively correlated with RSES scores at T2 (ρ = −0.357, *p* = 0.032) (Table 2). Figure 2 shows scatterplots of the correlations between scores for these variables in residents with MHP and residents without MHP.

**Table 2:** Correlations between GPIUS2 scores and other psychological variables in residents with MHP.

**Variable**	**General Health Questionnaire-12**		**Patient Health Questionnaire-9**		**Rosenberg Self-Esteem Scale**	
	**T1**	**T2**	**T1**	**T2**	**T1**	**T2**
	**ρ**	**ρ**	**ρ**	**ρ**	**ρ**	**ρ**
	**(*p*)**	**(*p*)**	**(*p*)**	**(*p*)**	**(*p*)**	**(*p*)**
GPIUS2 total score at T1	0.081	0.287	0.077	0.207	−0.099	−0.184
	(0.636)	(0.089)	(0.651)	(0.224)	(0.564)	(0.281)
GPIUS2 Preference for online social interaction at T1	−0.054(0.754)	0.131(0.443)	0.063(0.714)	0.051(0.766)	−0.035(0.835)	−0.134(0.434)
GPIUS2 Mood regulation at T1	−0.043	0.205	0.303	0.379	0.087	0.079
	(0.802)	(0.230)	(0.072)	(0.022^*^)	(0.613)	(0.646)
GPIUS2 Deficient self-regulation at T1	0.123	0.235	−0.026	0.067	0.006	−0.023
	(0.472)	(0.166)	(0.878)	(0.697)	(0.968)	(0.893)
GPIUS2 Negative outcomes at T1	0.056	0.276	−0.242	−0.050	−0.132	−0.167
	(0.744)	(0.103)	(0.153)	(0.772)	(0.441)	(0.328)
GPIUS2 total score at T2	0.020	0.133	0.135	0.286	−0.140	−0.260
	(0.903)	(0.437)	(0.430)	(0.090)	(0.413)	(0.125)
GPIUS2 Preference for online social interaction at T2	0.146	0.301	0.382	0.428	−0.304	−0.447
	(0.395)	(0.074)	(0.021^*^)	(0.009^**^)	(0.071)	(0.006^*^)
GPIUS2 Mood regulation at T2	−0.125	0.042	0.299	0.371	0.058	0.037
	(0.464)	(0.804)	(0.075)	(0.025^*^)	(0.734)	(0.828)
GPIUS2 Deficient self-regulation at T2	0.082(0.632)	0.002(0.987)	0.139(0.415)	0.224(0.187)	−0.025(0.883)	−0.223(0.190)
GPIUS2 Negative outcomes at T2	0.207	0.188	0.072	0.131	−0.197	−0.357
	(0.225)	(0.269)	(0.674)	(0.444)	(0.248)	(0.032^*^)

MHP, mental health problems; GPIUS2, Generalized Problematic Internet Use Scale 2; T1, Time 1 (baseline); T2, Time 2 (3 months after baseline);

* p < 0.05

** *p < 0.01*.

## Discussion

In the present study, we observed group differences between residents with MHP and those without MHP in GHQ, PHQ, RSES, and GPIUS2 scores. *Post-hoc* analyses showed that, compared with residents without MHP, those with MHP had higher GHQ scores at T2, higher PHQ scores at T1 and T2, and lower RSES scores at T1 and T2. These results suggest that signs of greater depression and lower self-esteem at the start of residency (i.e., T1) may be useful information for predicting which residents may exhibit MHP during the residency. Higher scores on the GPIUS2 subscale preference for online social interaction at T1 may also predict the potential emergence of MHP in residents. However, the information should be considered referential, as these scores in residents with MHP were generally within subclinical levels. The association between depression and self-esteem is well known; therefore, it would be useful to assess self-esteem along with depression in the mental health management of residents. Residents with no MHP may be able to cope with low levels of depression during the course of their residency, presumably because their experiences help to foster confidence and self-efficacy (24, 25). Confidence and self-efficacy are also associated with self-esteem, which we assessed using the RSES.

The observed pattern of greater Internet use in residents with MHP indicates that residents likely to develop future MHP have a tendency to engage in excessive Internet use during residency. Alternatively, Internet use could be interpreted as a coping behavior for stressful daily clinical practice. The between-group difference in GPIUS2 total scores and in scores on the subscales preference for online social interaction scores, mood regulation, and negative outcomes suggest that Internet use is a convenient index for detecting residents' MHP, together with conventional psychological measures of depression, anxiety, and self-esteem (12).

GPIUS2 mood regulation scores at T1 and T2 were positively correlated with PHQ depression scores at T2, indicating that a tendency to use the Internet for mood regulation predicts future depression in residents. Regarding GPIUS2 scores at T2 (but not T1), preference for online social interaction was positively correlated with PHQ depression scores at T1 and T2, and negatively correlated with self-esteem at T2 in residents with MHP. These associations show that highly depressed residents at baseline and 3-month follow-up tend to have higher Internet use, and indicate that depression level at baseline predicts the risk of greater Internet use for social interaction, and that current depression status reflects residents' Internet use. Furthermore, lower self-esteem at follow-up reflected a tendency for excessive Internet use, which is in line with the association between self-esteem and excessive Internet use found in previous studies (12, 26, 27). A possible interpretation of the preference for online social interaction in residents with MHP is that these individuals experience difficulties in face-to-face communication, particularly in medical settings. Alternatively, they may tend to use online communication tools such as social networking services for stress coping (12, 28, 29). Compared with residents without MHP, those with MHP scored higher on PHQ depression and lower on self-esteem at both T1 and T2. This indicates that residents with MHP had both a stable (trait) and more temporary state of depression and lower self-esteem, suggesting that they have difficulties coping with depression and fostering their own self-esteem throughout residency, which may lead to their higher levels of Internet use. Therefore, it may be possible (even for non-mental health expert educators) to manage residents' MHP by attempting to improve their depression and self-esteem to indirectly regulate their Internet use. This could be implemented through daily interaction between educators and learners, taking into account any difficulties experienced by residents in face-to-face communication. Interventions for comorbid psychiatric disorders are important for the treatment of excessive Internet use (30).

This study had several limitations. First, although this was a longitudinal study, the assumption of a causal relationship between Internet use and MHP must be made cautiously. Higher scores for a preference for online social interaction in residents with MHP at baseline indicate that Internet use may predict the later emergence of MHP. The positive correlation between mood regulation at baseline and depression at T2 also suggest the predictive nature of Internet use. However, the positive correlation between depression at baseline and preference for online social interaction at T2 indicate the inverse: that psychopathology can predict the degree of Internet use. Therefore, the present results should be considered only suggestive of a possible causal relationship between Internet use and mental health. Moreover, it is still unclear whether the higher Internet use in residents with MHP is addictive/problematic or is a compensatory coping reaction that has health benefits. Although low self-esteem at the follow-up was associated with the presence of Internet use negative outcomes in residents with MHP, the degree of Internet use was relatively low among participants. Therefore, future studies must recruit residents with heavy Internet use to clarify this point. Second, there were several missing data points because the submission rate was lower at T2 than at T1. Finally, there was no correction for multiple comparisons in the correlational analyses, because of the exploratory nature of the study.

In summary, we found that depression and low self-esteem were associated with higher Internet use 3 months after the start of residency, indicating that residents with a potential risk of MHP during the course of the residency may be at risk for excessive Internet use as a compensatory coping behavior. Depression and self-esteem at baseline may predict the level of Internet use at the time when the risk of MHP is highest for residents, although the assumption of a causal relationship between Internet use and mental health should be made with caution. It may be effective to provide interventions for residents with depression and low self-esteem to manage Internet use, which may help to avoid wasting societal resources by maintaining residents' mental health and their performance in clinical settings. Internet use may be a useful index of mental health for residents, as it is associated with depression, anxiety, and low self-esteem. It would be helpful to share information about residents' daily Internet use, particularly for educators who are not mental health experts.

## Data Availability Statement

The raw data supporting the conclusions of this article will be made available by the authors, without undue reservation.

## Ethics Statement

The studies involving human participants were reviewed and approved by Kyoto university graduate school and faculty of medicine, Ethics committee. The patients/participants provided their written informed consent to participate in this study.

## Author Contributions

TU and HF conceived, designed, and conducted the experiments, acquired and analyzed the data, and drafted the manuscript. HF contributed to the conception of the study, interpretation of data, and revisions for critically important intellectual content. TM and KI contributed to the design and data acquisition, interpretation of data, and drafting the manuscript. All authors approved the final manuscript for submission and agreed to be accountable for all aspects of the work, including the assurance that questions related to the accuracy or integrity of any part are appropriately investigated and resolved.

## Conflict of Interest

The authors declare that the research was conducted in the absence of any commercial or financial relationships that could be construed as a potential conflict of interest.
